# Extracellular vesicles released from hiPSC-derived MSCs attenuate chronic prostatitis/chronic pelvic pain syndrome in rats by immunoregulation

**DOI:** 10.1186/s13287-021-02269-x

**Published:** 2021-03-20

**Authors:** Xufeng Peng, Hailin Guo, Ji Yuan, Yu Chen, Yuguo Xia, Lin Wang, Ying Wang, Yichen Huang, Hua Xie, Yang Wang, Fang Chen

**Affiliations:** 1 Department of Urology, Shanghai Children’s Hospital, Shanghai Jiao Tong University, Shanghai, 200062 China; 2 Department of Urology, Shanghai Jiao Tong University Affiliated Sixth People’s Hospital, Shanghai, 200233 China; 3 Institute of Microsurgery on Extremities, Shanghai Jiao Tong University Affiliated Sixth People’s Hospital, Shanghai, 200233 China

**Keywords:** Mesenchymal stem cells, Extracellular vesicles, Chronic prostatitis/chronic pelvic pain syndrome, Immunoregulation

## Abstract

**Background:**

Chronic prostatitis/chronic pelvic pain syndrome (CP/CPPS) is an intractable nonbacterial inflammatory disease. Mesenchymal stem cells (MSCs) derived from human induced pluripotent stem cells (hiPSCs, iMSCs) have been well documented for the management of inflammatory and autoimmune disorders because of their powerful immunoregulatory and anti-inflammatory capacities. Recently, studies have indicated that extracellular vesicles (EVs) released from iMSCs hold biological functions similar to their parental cells. This study aimed to evaluate the therapeutic efficacy of EVs released from iMSCs (iMSCs-EVs) on CP/CPPS and to explore the underlying mechanisms.

**Methods:**

An experimental autoimmune prostatitis (EAP) model was established in rats by subcutaneous injection of prostate antigen with adjuvant. Then, iMSCs-EVs were injected into EAP rats via the tail vein. Pain behavioral measurements, urodynamic tests, and histopathological analyses were performed at 2, 4, and 6 weeks. The expression of cyclooxygenase-2 (COX-2) was evaluated by immunofluorescence staining and Western blot. The alterations of B cells, Th1 cells, Th2 cells, Th17 cells, and Treg cells in peripheral blood and spleen were analyzed using flow cytometry. The levels of Th1-, Th2-, Th17-, and Treg-related inflammatory mediators were determined by ELISA.

**Results:**

After iMSCs-EVs administration, rats had reduced pain as indicated by the recovery of nociceptive responses to baseline. The voiding pressure was significantly reduced, and the intercontraction interval was increased. The findings of histopathological analysis revealed that iMSCs-EVs could significantly decrease inflammatory cell infiltration and promote basal lamina and glandular epithelial tissue repair. Further studies demonstrated that the overexpression of COX-2 was downregulated by iMSCs-EVs. Meanwhile, the increases in the percentages of Th1 and Th17 cells were dramatically reversed. Also, rats that received iMSCs-EVs showed markedly increased percentages of Treg cells. The levels of those inflammatory mediators showed the same changing tendency.

**Conclusions:**

iMSCs-EVs administration has the potential to ameliorate chronic pelvic pain, improve voiding dysfunction, suppress inflammatory reactions, and facilitate prostatic tissue repair. The functions are mediated by downregulating the overexpression of COX-2 and restoring the imbalance of Th1/Th2 and Treg/Th17 cells.

**Supplementary Information:**

The online version contains supplementary material available at 10.1186/s13287-021-02269-x.

## Background

Chronic prostatitis/chronic pelvic pain syndrome (CP/CPPS) remains the most challenging andrological dilemma in male patients younger than 50 years, accounting for more than 90–95% of prostatitis diagnoses [1, 2]. Clinically, this type of prostatitis is defined as chronic pelvic pain and symptoms of prostate inflammation lasting at least 3 to 6 months in the absence of any detectable infectious agents [3]. It is characterized by chronic pain in the region of pelvic, often accompanied by voiding dysfunction and sexual complaints [4]. The symptoms associated with CP/CPPS have a markedly negative effect on quality of life that is comparable to those suffering from myocardial infarction or Crohn’s disease [5]. To date, there is no specific treatment. Thus, it is imperative to develop an effective therapeutic strategy.

Although multiple factors including imbalance of hormones, intraprostatic urinary reflux, nervous system, and psychosocial conditions have been involved in triggering CP/CPPS [6], accumulating evidence from clinical trials has shown that this syndrome is a consequence of dysregulated inflammation associated with autoimmune disorders. Indeed, elevated levels of IgG autoantibodies and proinflammatory mediators have been detected in serum and prostatic secretions from CP/CPPS patients, and the proliferative response of CD4+ T cells has also been observed [7, 8]. According to these findings, rodent models of experimental autoimmune prostatitis (EAP) which can mirror the clinical, immunological, and pathological characteristics of human CP/CPPS, have been successfully established by subcutaneous injection of prostate antigen with adjuvant and are deemed valid tools to explore the mechanisms and possible therapies of CP/CPPS [9, 10].

Currently, mesenchymal stem cells (MSCs) have been well documented for the management of inflammatory and autoimmune disorders because of their powerful immunoregulatory and anti-inflammatory capacities [11, 12]. As an alternative source of stem cells, human induced pluripotent stem cells (iPSCs) possess unlimited self-renewal and differentiation capacity which could provide a large amount of cells [13]. We and other research groups have recently derived MSC from iPSCs [14, 15], providing a new source of MSCs (iMSCs). iMSCs have been proven to be alike adult MSCs in morphology, global gene expression, and function [16]. Moreover, when compared with adult MSCs, human iMSCs have been demonstrated to be superior with regard to cell proliferation, immunomodulation, and capability of modulating the microenvironment [17, 18]. Recently, studies have indicated that small extracellular vesicles (EVs) released from iMSCs have biological functions similar to those of their parental cells [19]. EVs (30–150 nm in diameter) [20] represent an exciting, potentially multitarget therapeutic for autoimmune disorders. They are endosomal-origin nanosized liposomes and contain various functional biochemicals, such as mRNAs, proteins, and microRNAs, that play critical roles in intercellular communication [21]. Most importantly, the ingredients of EVs are protected by the lipid-bilayer from extracellular destruction. Transplantation of EVs avoids the potential risks of direct stem cell transplantation, including abnormal differentiation, tumorigenicity, and vascular blockage [22]. Therefore, EVs released from MSC are emerging as an extremely promising cell-free strategy for treating autoimmune disorders.

Our group previously demonstrated that EVs released from MSCs derived human induced pluripotent stem cells (hiPSCs, iMSCs-EVs) exerted stronger therapeutic benefits on the treatment of osteoarthritis than do synovial membrane-derived MSCs [17]. In addition, Fang et al. [23] reported that iMSCs were the ideal cellular source for the large-scale production of EVs and confirmed that iMSCs-EVs could attenuate allergic airway inflammation. These inspiring findings suggest that the transplantation of iMSCs-EVs might be useful for the management of CP/CPPS. In the present study, we investigated the therapeutic effect of iMSCs-EVs on CP/CPPS and explored the potential mechanisms of immunoregulatory properties in a rat EAP model. Herein, this work first demonstrated that iMSCs-EVs could significantly ameliorate chronic pelvic pain, improve voiding dysfunction, suppress inflammatory reactions by downregulating the overexpression of cyclooxygenase-2 (COX-2), and restore the imbalance of Th1/Th2 and Th17/Treg cells.

## Materials and methods

All animal experiments were approved by the local ethics committee of Shanghai Jiao Tong University (approval code: DWSY2018-151).

### Cell culture and identification

The hiPSC lines were provided by the Institute of Biochemistry and Cell Biology of the Chinese Academy of Sciences. Three hiPSC lines (iPS-S-01, C1P33, and PCKDSF001C1) were utilized to induce iMSCs as previously described [24]. Briefly, vitronectin (Nuwacell™, cat.no. RP01002) was used to precoat a 6-well plate (1 μg/cm^2^) at 25 °C for 2 h. Subsequently, hiPSC were seeded in serum-free ncEpi basal medium (Nuwacell™, cat.no. RP01001) in a 6-well plate. After cells were cultured to 90% confluence, the culture medium was replaced by serum-free ncMission basal medium (Nuwacell™, cat.no. RP0201-01) and supplement (Nuwacell™, cat.no. RP02010-02). The cell culture medium was changed at 2–3 day intervals. The cells were separated using 0.25% trypsin (Gibco, cat.no.25200056) and reseeded in 75cm^2^ canted neck flasks (Corning, NY). When cells were cultured to 85–90% confluence, they were regarded as passage 1. Cells at passage 5 usually showed typical fibroblastic morphology and were collected to identify iMSCs phenotypic characteristics, and differentiation potentials. Passages 6 to 9 iMSCs were utilized for the following experiments.

The phenotypes of iMSCs were analyzed by flow cytometry. A single-cell suspension was obtained by trypsinizing and 3% bovine serum albumin (BSA, Gibco, cat.no.16140063) was utilized to block nonspecific antigen binding. Then, iMSCs were incubated with the following monoclonal antibodies (eBioscience,San Diego, USA): CD11b-PE, CD19-FITC, CD34-APC, CD44-PE, CD45-APC/Cyanine7, CD73-FITC, CD90-PE, CD105-PE, and HLA-DR-PerCP-Cyanine. To detect osteogenic and adipogenic differentiation, cells at passage 5 were incubated separately with osteogenesis medium (Gibco, cat.no. A1007201) and adipogenesis medium (Gibco, cat.no. A1007001) following the supplier’s instructions for 3 weeks. The induced cells were identified by Oil Red O and Alizarin Red staining, respectively. To prove that iMSCs are not contaminated with fibroblastic cells, immunofluorescent staining for vimentin was performed.

### Isolation and purification of iMSCs-EVs

iMSCs-EVs were isolated and purified by differential ultracentrifugation as previously reported [25]. Serum-free/xeno-free conditioned medium (CM) was collected when iMSCs were cultured to 85–90% confluence. The obtained CM was differentially centrifuged at 300×*g* for 20 min, and 2000×*g* for 15 min at 4 °C, and then filtered using a 0.22-μm sterilized filter (Thermo Scientific, cat.no.42225-PV) to eliminate dead cells and cellular debris. The CM was further ultracentrifuged at 100,000×*g* for 120 min at 4 °C using a Beckman ultracentrifuge (Beckman Coulter, Germany). The ice-cold phosphate-buffered saline (PBS) was utilized to wash the pelleted EVs three times. Finally, the EVs were resuspended in PBS and stored at − 80 °C for the following experiments.

### Characterization of iMSCs-EVs

The morphology of iMSCs-EVs was identified using transmission electron microscopy (TEM). Three precent (w/v) glutaraldehyde was utilized to fix the pelleted iMSCs-EVs. After washing, they were stained with 2% uranyl acetate and visualized by TEM (H7650; HITACHI, Japan). The size distribution and particle concentration of iMSCs-EVs were measured using a flow nanoanalyzer (NanoFCMlnc, Xiamen, China) as previously reported [26]. The specific markers of iMSCs-EVs were confirmed by classical Western blot, and the markers included CD63 (1:300, Abcam), Alix (1:300, Cell Signaling Technology), and TSG101 (1:300, Abcam). The expression of cis-Golgi matrix protein GM130 (1:300, Abcam) was detected to evaluate the purity of iMSCs-EVs [26].

### EAP model induction and iMSCs-EVs administration

Thirty-five adult male Sprague-Dawley (SD) rats (180–200 g) were purchased from Shanghai Experimental Animals Center (Shanghai, China). Eight rats were utilized to prepare prostate antigen homogenate supernatant (PAHS). Twenty-seven rats were randomly divided into three groups: the control group (treated with an equal volume of PBS, *n* = 9), the model group (received PAHS to introduce EAP and treated with an equal volume of PBS, *n* = 9), and iMSCs-EVs group (received PAHS to introduce EAP and treated with iMSCs-EVs). The EAP model was induced as previous reports [9]. Briefly, the rats were immunized on days 0, 15, and 30 with 1.0 ml of PAHS emulsified with complete Freund’s adjuvant by multipoint subcutaneous injection, meanwhile, 0.5 ml pertussis–diphtheria-tetanus vaccine was administered by intraperitoneal injection. In the iMSCs-EVs group, rats were treated with iMSCs-EVs (1 × 10^10^ particles in 200 ul PBS) via tail vein injection once a week. At 2, 4, and 6 weeks, animals were euthanized, and blood, spleen, and prostate were harvested for analysis.

### Pain behavioral measurement

The animals were brought into a quiet laboratory. The tests were carried out in individual acrylic cages on a wire grid floor. Tactile allodynia over the area near the prostate was assessed by the Electronic Von Frey (BIO-EVF4-S; Bioseb) at 2, 4, and 6 weeks. Starting from the lowest force, animals were stimulated with forces of 0.4, 1, and 4 g. The stimulation was repeated until the animal presents five similar measurements. A positive response was defined by the following behaviors: jumping, immediate scratching or licking of the stimulated region, and sharp contraction of the lower abdomen.

### Urodynamic test

The urodynamic test was performed as previously reported [27]. After anesthetized with urethane (1.2 g/kg, intraperitoneal), a lower midline abdominal incision was made to expose the urinary bladder. A polyethylene catheter (PE-50) with a flared end was inserted through the vesical dome, and the other end of this catheter was connected via a 3-way stopcock to a microinfusion pump and a pressure transducer (AD Instruments, Australia). Continuous vesical infusion with normal saline was performed at a flow rate of 4 ml/h. The urodynamic parameters recorded were maximal voiding pressure (cmH_2_O) and intercontraction interval (second).

### Inflammatory mediators measurement in serum

Blood samples were obtained from the tail vein. After blood coagulation, it was then centrifuged at 4000 rpm for 15 min at 4 °C.The protein concentrations of inflammatory mediators (IFN-γ [interferon-γ], IL-2 [interleukin-2], IL-4 [interleukin-4], IL-6 [interleukin-6], IL-10 [interleukin-10], and IL-17A [interleukin-17A]) were measured using commercial enzyme-linked immunosorbent assay (ELISA) kits (Abcam, Cambridge), following the manufacturer’s protocols.

### Flow cytometry for B cells and T cells

Peripheral blood and spleen samples were obtained from rats and single-cell suspensions were prepared in Cell Staining Buffer (Biolegend, cat.no.420201). The single-cell suspensions were centrifuged at 350*g* for 5 min at 4 °C. To remove red blood cells, 1 × Red Blood Cell Lysis Buffer (Biolegend, cat.no.420301) was utilized to resuspend the pellet. Subsequently, surface markers were stained with fluorescence-labeled antibodies (APC-anti-CD3, APC/Cyanine7-anti-CD4, PE/Cyanine7-anti-CD45RA). For nuclear staining (PE-anti-Foxp3), cells were permeabilized with the Nuclear Factor Fixation and Permeabilization Buffer Sets (Biolegend, cat.no.422601). For intracellular staining (FITC-anti-IFN-γ, PE-anti-IL-4, PerCP-Cyanine5.5-anti-IL-17A), cells were treated with Cell Activation Cocktail (Biolegend, cat.no.423304). All antibodies were purchased from Abcam (Cambridge, UK). Finally, the stained cells were analyzed by an LSRII flow cytometer (BD Biosciences).

### Calculation of the prostate index

Rats were euthanized at 2, 4, and 6 weeks, and prostate samples of all rats were collected and weighed. Then, the prostate index was calculated as follows: prostate index = (prostate weight/rat weight) × 10,000.

### Histopathological analysis

Four percent paraformaldehyde was utilized to fix the prostatic tissues for 48 h. Then the samples were placed in ethanol to dehydrate and then embedded in paraffin wax. Next, they were sliced into 5-μm-thick sections and stained with hematoxylin and eosin (H&E) for histopathological examination. A 4-point grading scale [1] was adopted to evaluate the degree of inflammation in prostatic tissues: 0, no inflammation; 1, mild but definite perivascular cuffing with mononuclear cells; 2, moderate perivascular cuffing with mononuclear cells; 3, marked perivascular cuffing, hemorrhage with some parenchymal inflammatory cells; and 4, marked perivascular cuffing, hemorrhage, and numerous mononuclear and mast cells in the parenchyma.

To identify the expression of COX-2 in prostatic tissues, immunofluorescence staining was conducted using an antibody against COX-2 (Abcam, cat.no.ab15191). To investigate the status of immune cells (B cells and T cells) that infiltrate in the prosate tissues, immunohistochemical staining was perfomed. Images were captured by a fluorescence microscope (Carl Zeiss, Jena, Germany). Image-Pro Plus 5.0 (Bethesda, USA) was utilized to analyze the positively stained cells. Eight different fields for each section were utilized for quantification.

### Western blot analysis

Rat prostatic samples were homogenized. Total proteins were harvested using a commercial Protein Extraction Kit (Yeasen Biotech, cat.no.20126ES60). The protein amounts were measured using a BCA Protein Assay Kit (Beyotime Biotechnology, China). Equal aliquots of sample extracts were loaded into 12% sodium dodecyl sulfate-polyacrylamide gels. After proteins were separated, they were transferred to polyvinylidene difluoride (PVDF) membranes. Nonfat milk (5%) was utilized to block the nonspecific binding. Then, the membranes were incubated for 12 h at 4 °C with antibodies against COX-2 (1:500; Abcam), and GAPDH (1:500; Abcam). The ECL (enhanced chemiluminescence, USA) technique was utilized to expose the protein bands.

### Statistical analysis

Statistical tests were carried out using GraphPad Prism 8 (GraphPad, Software). Experimental data are shown as the mean ± SD (standard deviation). One-way analysis of variance (ANOVA) was utilized to compare differences among different groups. A value of *P* < 0.05 was taken to indicate significance.

## Results

### Characterization of iMSCs

**Fig. 1:**
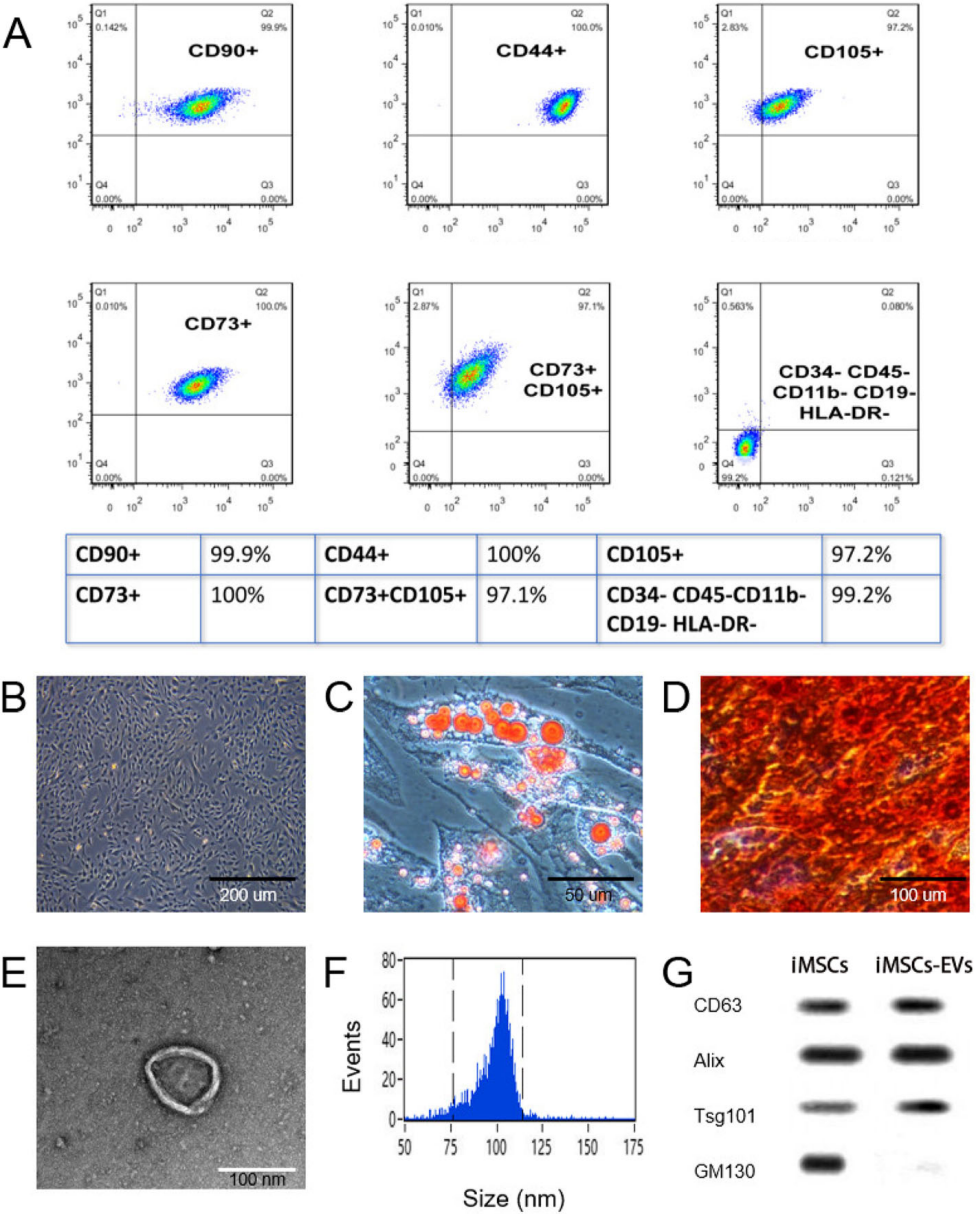
Identification of iMSCs and iMSCs-EVs. **a** Flow cytometric analysis demonstrated that iMSCs strongly expressed characteristic surface markers including CD44, CD73, CD90, and CD105, but not CD11b, CD19, CD34, CD45, and HLA-DR-. **b** The morphology of iMSCs. **c** The adipogenic differentiation of iMSCs. **d** The osteogenic differentiation of iMSCs. **e** Morphology of iMSCs-EVs under TEM. **f** The particle size distribution of iMSCs-EVs analyzed by Flow nano-analyzer. **g** Western blotting analysis showed that iMSCs-EVs positively expressed the specific markers CD63, Alix, and Tsg101, while the cis-Golgi matrix protein GM130 was negative

After 5 times of passage, iMSCs were successfully induced from iPSCs. To identify the phenotype of iMSCs, flow cytometry analysis was performed. The findings demonstrated that iMSCs strongly expressed pluripotency-related surface markers including CD44, CD73, CD90, and CD105, but not CD11b, CD19, CD34, CD45, and HLA-DR- (Fig. 1a). The expanded iMSCs displayed homogeneous spindle-shaped morphology under an optical microscope (Fig. 1b), which is in line with our previous report [17]. Furthermore, adipogenic and osteogenic differentiation assays were performed to examine the multipotent differentiation potential, and the results showed that iMSCs could be induced into cells positively stained with Oil Red O (Fig. 1c) and Alizarin Red (Fig. 1d), respectively. Additionally, immunofluorescent results showed that iMSCs did not express vimentin (Fig. 1s). Thus, iMSCs were not contaminated with fibroblasts.

### Characterization of iMSCs-EVs

iMSCs-EVs were successfully isolated by differential ultra-centrifugation. TEM images revealed that iMSCs-EVs exhibited cup-shaped morphology (Fig. 1e). Nanoflow findings confirmed that the diameters of iMSCs-EVs were 50–150 nm and the particle concentration was approximately 1.35 × 10^11^ particles/ml (Fig. 1f). The results of Western blot indicated that iMSCs-EVs positively expressed the specific markers CD63, Alix, and Tsg101, but the cis-Golgi matrix protein GM130 was negative (Fig. 1g).

### iMSCs-EVs ameliorate the nociceptive response

**Fig. 2:**
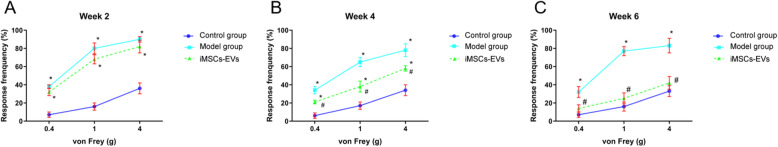
Pain behavioral measurement. The response frequencies of the three groups under mechanical stimulation with forces of 0.4, 1.0, and 4.0 g at 2 (**a**), 4 (**b**), and 6 (**c**) weeks. Data are presented as mean ± SD, *P < 0.05 compared with the control group, ^#^*P* < 0.05 compared with the model group

Measurements from the electronic Von Frey tests were utilized to assess the effect of iMSCs-EVs on tactile allodynia. Nociceptive responses were monitored for the three groups at 2, 4, and 6 weeks. (Fig. 2a–c). Rats in the model group displayed significant increases in response frequencies compared with rats in the control group (*P* < 0.05). Although the response frequencies showed no difference between the model and iMSCs-EVs groups at 2 weeks (*P* > 0.05), iMSCs-EVs administration significantly reduced the response frequencies with forces of 0.4, 1.0, and 4.0 g at 4 weeks (*P* < 0.001). At 6 weeks, the response frequencies of rats in the iMSCs-EVs group were reduced to baseline and were similar to those of the control group (*P* > 0.05).

**Fig. 3:**
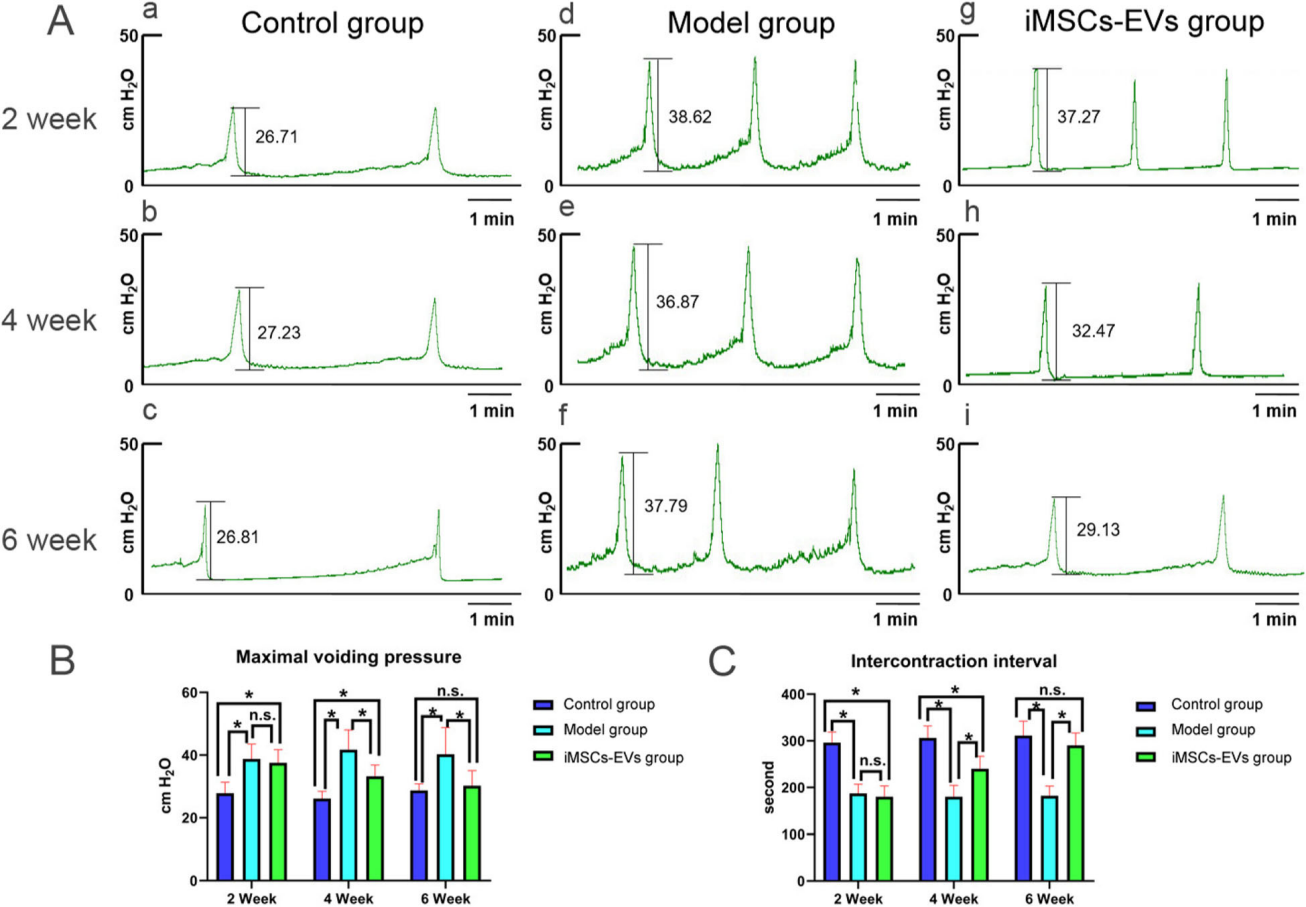
Assessment of voiding function. **A** Cystometrography curves of the different groups at 2, 4, and 6 weeks. **B**, **C** Statistical results of maximal voiding pressure and intercontraction interval showed that iMSCs-EVs administration significantly reduced the voiding pressure and the increased intercontraction interval at 4 weeks. These parameters reached a normal level at 6 weeks. Data are presented as the mean ± SD, *P < 0.05, n.s. indicates no significance

### iMSCs-EVs improve the urodynamic parameters

Voiding dysfunction is one of the principal manifestations of CP/CPPS. To test the therapeutic effect of iMSCs-EVs in this respect, the maximal voiding pressure and intercontraction interval of rats in different groups were measured and analyzed (Fig. 3). At 2 weeks, the model group had a significantly higher maximal voiding pressure and a shorter intercontraction interval than the control group (37.3 ± 2.8 cmH_2_O vs 24.5 ± 2.3 cmH_2_O; 167.5 ± 26.4 s vs 276.2 ± 28.8 s, respectively, *P* < 0.001). No significant improvement in maximal voiding pressure and intercontraction interval was observed in the iMSCs-EVs group compared with the model group (35.3 ± 3.1 cmH_2_O vs 37.3 ± 2.8 cmH_2_O; 266.7 ± 31.6 s vs 276.2 ± 28.8 s, respectively, *P* > 0.05). However, after 4-week posttreatment, significant improvements in maximal voiding pressure and intercontraction interval were noted in the iMSCs-EVs group compared with the model group (30.3 ± 2.3 cmH_2_O vs 36.3 ± 2.7 cmH_2_O; 221.7 ± 27.2 s vs 276.2 ± 28.8 s, respectively, *P* < 0.05). After 6-week posttreatment, these parameters showed no difference between the iMSCs-EVs and control groups (27.8 ± 2.4 cmH_2_O vs 25.5 ± 2.7 cmH_2_O; 274.9 ± 22.3 s vs 278.8 ± 21.6 s, respectively, *P* > 0.05). These data suggested that iMSCs-EVs could make the completely recover the maximal voiding pressure and intercontraction interval.

### iMSCs-EVs decrease the prostate index

**Fig. 4:**
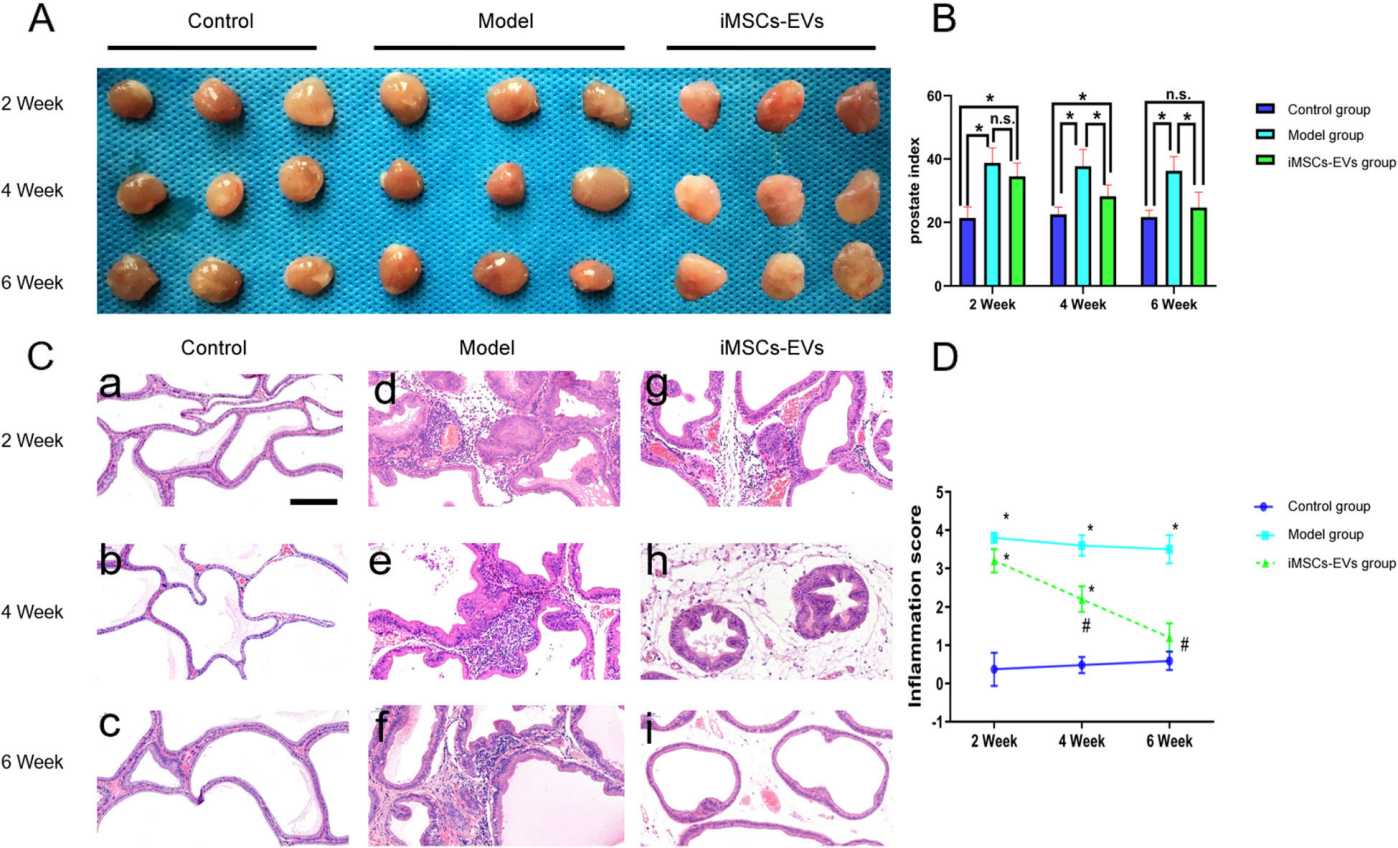
iMSCs-EVs administration decreased the prostate index and promoted prostatic tissue repair. **A** Images of prostatic tissue in each group at the indicated times. **B** Prostate indexes were calculated for the three groups. **C** Representative H&E sections of prostates from the three groups. Scale bar = 200 μm. **D** Inflammation scores for the three groups were analyzed. Data are presented as the mean ± SD, *P < 0.05 compared with the control group, ^#^*P* < 0.05 compared with the model group. n.s. indicates no significance

The prostate index is a frequently used indicator that increases with the degree of severity of prostatic inflammation. After the rat EAP model was induced, marked increases were found in the prostate index. Although this indicator did not improve in the iMSCs-EVs group compared with the model group at 2 weeks (38.1 ± 3.2 vs 40.2 ± 2.7, *P* = 0.67 > 0.05), marked decreases were observed after 4 weeks (28.3 ± 3.4 vs 39.4 ± 3.1, *P* = 0.038 < 0.05), and 6 weeks (22.4 ± 2.7 vs 38.8 ± 3.5, *P* < 0.001) iMSCs-EVs administration (Fig. 4A, B). These results indicated that iMSCs-EVs could alleviate prostatic hyperemia and edema.

### iMSCs-EVs attenuate histological damage

Histological analysis, which can accurately reflect the degree of EVserity of prostatitis, is the most direct evaluation criterion. In the control group, there were no clues of inflammatory alterations in the prostatic tissues (Fig. 4C-a, C-b, C-c). In contrast, the destruction of glandular epithelial tissue and basal lamina, infiltration of inflammatory cells around the periglandular tissue and in the stromal tissue, and interstitial edema were observed in the model group (Fig. 4C-d, C-e, C-f). At the early time point of 2 weeks, noticeable improvements in histopathological characteristics (Fig. 4C-g) and inflammation scores (3.32 ± 0.41 vs 3.82 ± 0.23, *P* = 0.089 > 0.05) did not display in the iMSCs-EVs group compared with the model group. After 4-week posttreatment, notably, decreased inflammatory cell infiltration, improved basal lamina, glandular epithelial tissue (Fig. 4C-h) and decreased inflammation scores (2.24 ± 0.32 vs 3.63 ± 0.34, *P* = 0.037 < 0.05) were observed in the iMSCs-EVs group when compared with the model group. After 6-week posttreatment, the iMSCs-EVs group showed marked restoration of the acinar histoarchitecture with only sporadic inflammatory cells in the stromal tissue (Fig. 4C-i). Accordingly, the iMSCs-EVs group had an inflammation score of 1.15 ± 0.32, which was lower than that of the model group (3.46 ± 0.47,*P* = 0.013 < 0.05) and was comparable to that of the control group (0.86 ± 0.42, *P* = 0.67 > 0.05) (Fig. 4D). Taken together, our findings demonstrated that iMSCs-EVs could improve prostatitis by suppressing the inflammatory reaction and promoting prostatic tissue repair.

### iMSCs-EVs downregulate the overexpression of COX-2

**Fig. 5:**
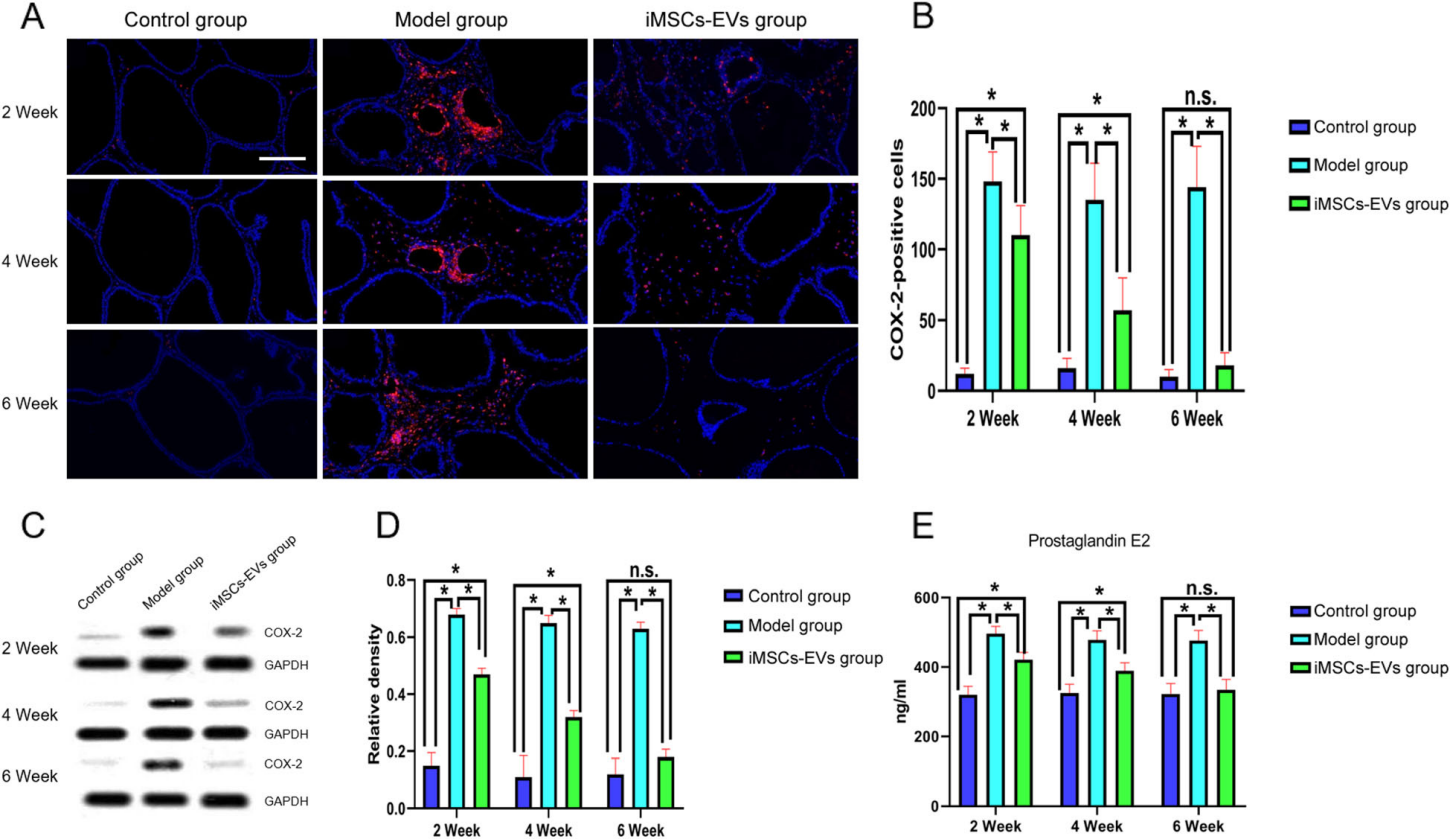
iMSCs-EVs administration downregulated the overexpression of COX-2 and prostaglandin E2. **a** Representative fluorescence images of COX-2 expression in prostatic tissues. COX-2^+^ cells were stained red. Nuclei were stained blue. Scale bar = 200 μm. **b** Statistical results of the numbers of COX-2^+^ cells. **c**, **d** Western blotting was utilized to quantify COX-2 protein levels in prostatic tissues. **e** The levels of prostaglandin E2 were determined by ELISA. Data are presented as the mean ± SD, *P < 0.05, n.s. indicates no significance

In the course of CP/CPPS, the overexpression of COX-2 and its downstream prostaglandin E2 play the main roles in the production of pain. To study the antalgic mechanism of iMSCs-EVs, our group investigated the expression of COX-2 in prostatic tissue using immunofluorescence staining and western blot. Next, the levels of prostaglandin E2 were detected by ELISA. As shown in Fig. 5, the levels of COX-2 were markedly upregulated in the model group compared to that of the control group. Nevertheless, after iMSCs-EVs administration, the COX-2 protein level was sharply decreased. Consistent with the changes in COX-2, the results of ELISA analysis showed that the decreases in COX-2 downregulated the production of prostaglandin E2 (Fig. 5e). These results illuminated that iMSCs-EVs could ameliorate chronic pelvic pain by downregulating the overexpression of COX-2.

### iMSCs-EVs correct the imbalance of Th1/Th2 and Treg/Th17 cells

**Fig. 6:**
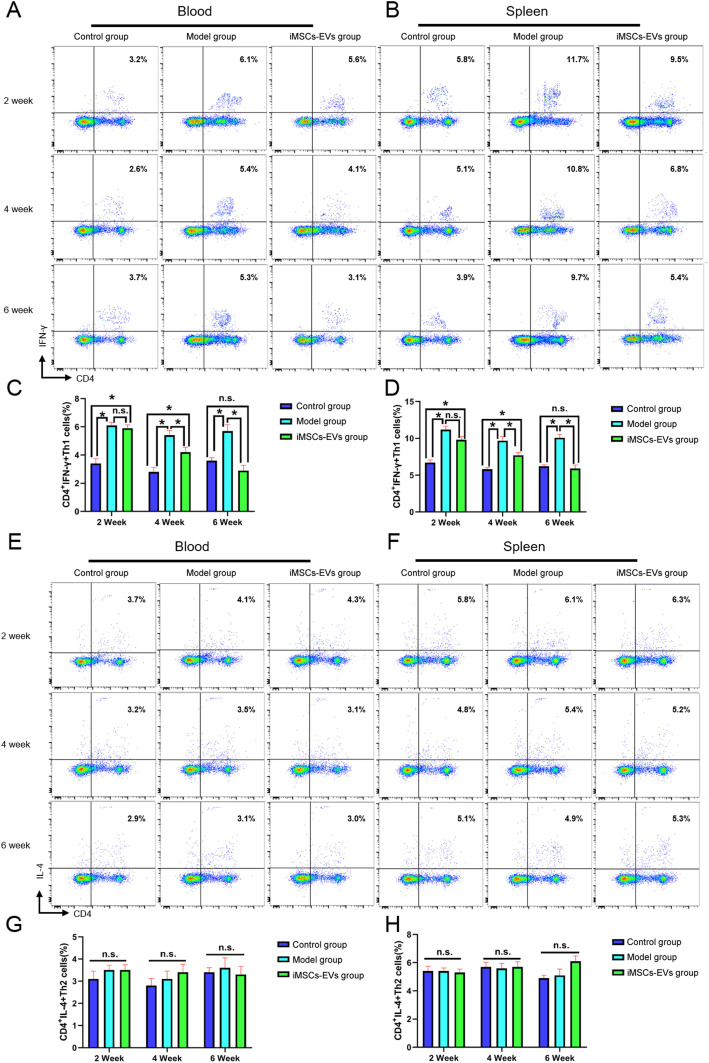
Effect of iMSCs-EVs administration on the percentages of Th1 and Th2 cells in peripheral blood and spleen. **a**, **b**, **e**, and **f** Representative flow cytometric plots in each group. **c**, **d** Statistical results indicated that iMSCs-EVs administration could reverse the increases in the percentages of Th1 cells. **g**, **h** Statistical results indicated that no difference existed in the percentages of Th2 cells among the three groups. Data are presented as the mean ± SD, *P < 0.05, n.s. indicates no significance

**Fig. 7:**
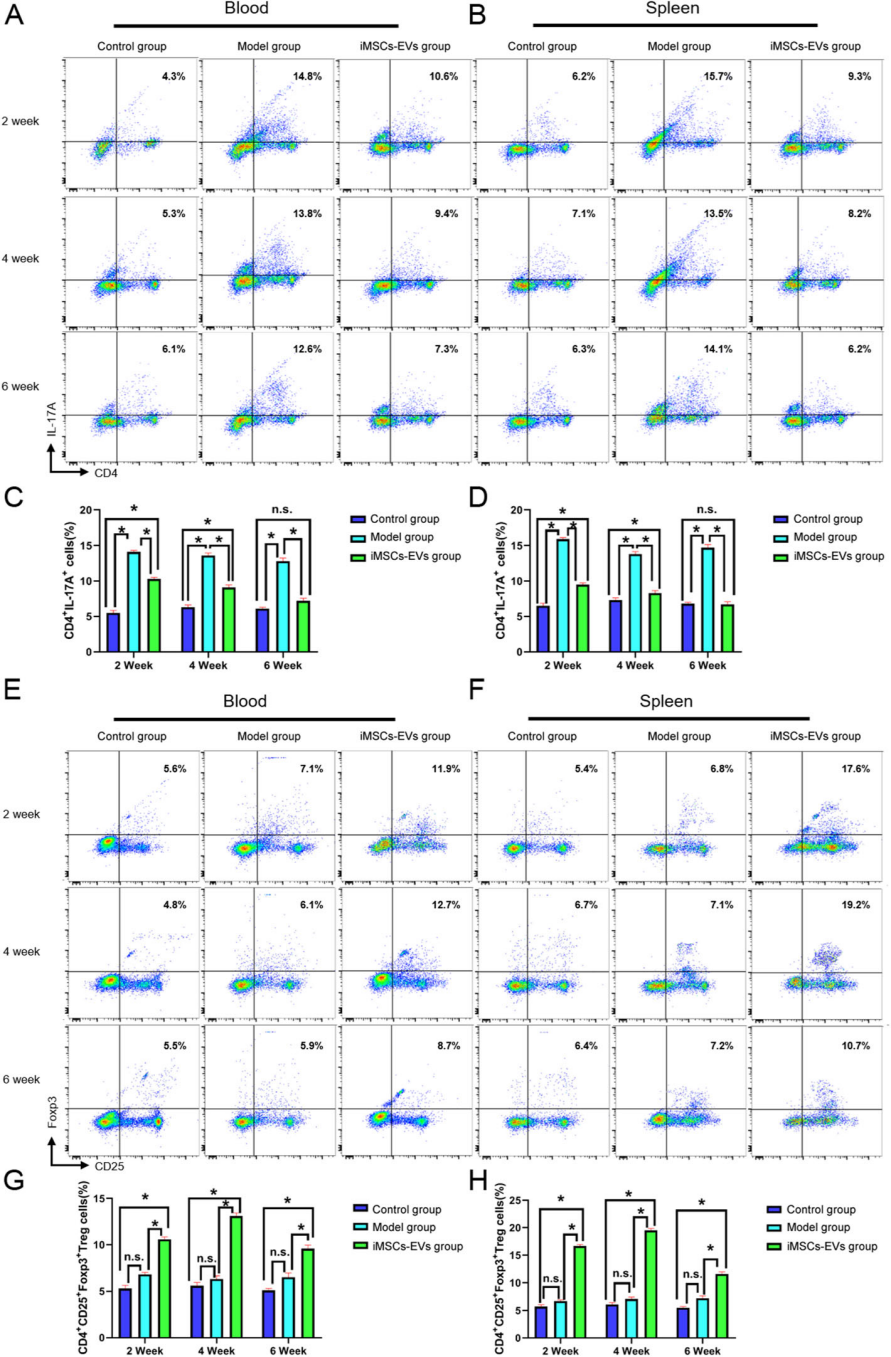
Effect of iMSCs-EVs administration on the percentages of Th17 and Treg cells in peripheral blood and spleen. **a**, **b**, **e**, and **f** Representative flow cytometric plots in each group. **c**, **d** Statistical results indicated that iMSCs-EVs administration could increase the percentages of Treg cells. **g**, **h** Statistical results indicated that iMSCs-EVs administration could reverse the increases in the percentages of Th17 cells. Data are presented as the mean ± SD, *P < 0.05, n.s. indicates no significance

**Fig. 8:**
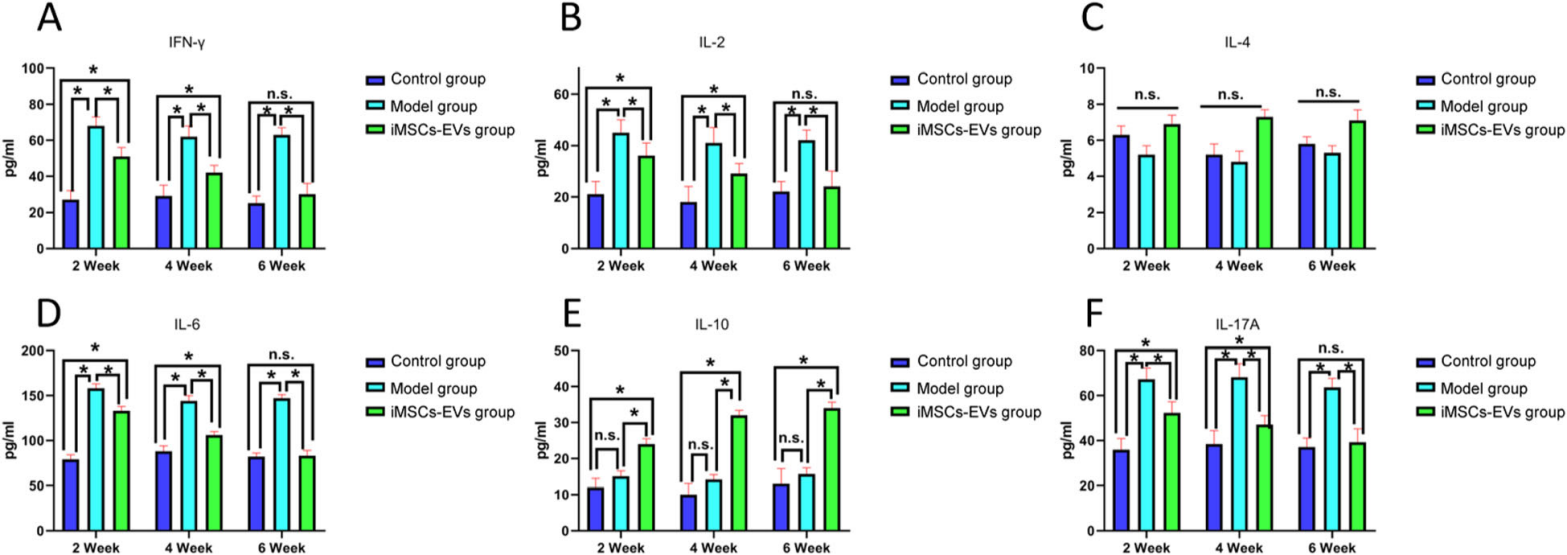
Quantitative analysis of Th1-, Th2-, Th17-, and Treg-related inflammatory mediators in the three groups. The relative protein expression levels of IFN-γ (**a**), IL-2 (**b**), IL-4 (**c**), IL-6 (**d**), IL-10 (**e**), and IL-17A (**f**). Data are presented as the mean ± SD, *P < 0.05, n.s. indicates no significance

To further explore the underlying mechanism of the immunoregulatory effects of iMSCs-EVs, the alterations of CD3^−^CD45RA^+^B cells, CD4^+^IFN-γ^+^Th1cells, CD4^+^IL-4^+^Th2 cells, CD4^+^IL-17A^+^Th17cells, and CD4^+^CD25^+^Foxp3^+^Treg cells in peripheral blood and spleen were analyzed using flow cytometry. At 2, 4, and 6 weeks, there were no obvious alterations in the percentages of B cells (Fig. S2) and Th2 cells (Fig. 6e–h) among the three groups (*P* > 0.05). Relative to the control group, the higher percentages of Th1 (Fig. 6a–d) cells and Th17 (Fig. 7a–d) cells were observed in the model group at the three time points. Although the percentage of Th1 cells was not downregulated in the iMSCs-EVs group compared with the model group at 2 weeks, decreases in the percentages of Th1 cells were displayed at 4 and 6 weeks. The increases in the percentages of Th17 cells were downregulated in the iMSCs-EVs group at 2 and 4 weeks and restored to normal levels at 6 weeks. In addition, rats in the iMSCs-EVs group showed that the percentages of Treg cells (Fig. 7e–h) were markedly increased at the three time points (*P* < 0.05). To investigate the status of immune cells that infiltrate in the prostate tissues, alterations of the aforementioned immune cells were evaluated by immunohistochemical staining. The same change trends were observed in the prostate tissues (Fig. S3–5). Furthermore, Th1-, Th2-, Th17-, and Treg-related inflammatory mediators were measured by ELISA (Fig. 8), and consistent results were observed. The levels of IFN-γ and IL-2 (secreted by Th1 cells) and IL-6 and IL-17A (secreted by Th17 cells) were significantly upregulated in the model group (*P* < 0.05), while iMSCs-EVs dramatically reduced the expression of these four proinflammatory mediators and upregulated the expression of the anti-inflammatory mediator IL-10 (secreted by Treg cells). Collectively, these findings illustrated that iMSCs-EVs could restore the imbalance of Th1/Th2 and T17/Treg by decreasing the percentages of Th1 cells and Th17 cells and increasing the percentage of Treg cells.

## Discussion

In clinical practice, current therapies for CP/CPPS are only limited to relieve symptoms [28]. The available options include antibiotics, analgesics, α-receptor blockers, and neuroleptics [29]. The therapeutic effect is far from satisfactory for either sufferers or urologists. In this study, we confirmed that iMSCs-EVs administration could effectively ameliorate chronic pelvic pain, reduce voiding pressure, increase the intercontraction interval, suppress inflammatory reactions and promote prostatic tissue repair. Further studies revealed that iMSCs-EVs administration could downregulate the overexpression of COX-2 and restore the imbalance of Th1/Th2 and Treg/Th17 cells. These results provide a brand-new and cell-free strategy for the management of CP/CPPS.

For CP/CPPS patients, the most prominent symptom is recurrent and long-term pain in the genitals, perineum, lower back, and even during or after ejaculation [30]. Patients who suffer from chronic pain may experience anxiety, depression, and other unfavorable mental conditions. Both clinical studies and animal experiments have demonstrated that prostaglandin E2 induced by COX-2 could sensitize peripheral nociceptors through the activation of prostaglandin E2 receptors [31]. This sensitization, leading to a reduction in the nociceptive threshold, is the main characteristic of inflammatory hyperalgesia in the peripheral tissue [11]. In the development of CP/CPPS, elevated levels of COX-2 and prostaglandin E2 were detected and proved to play critical roles in the generation of hyperalgesia [32]. Therefore, if there are strategies to downregulate the overexpression of COX-2, chronic pain will be ameliorated. Recently, Orozco et al. [33] documented a clinical study in which 10 patients diagnosed with discogenic back pain received autologous MSCs and showed rapid improvement in disability and pain. More recently, Shiue et al. [34] reported that EVs released from umbilical cord MSCs could inhibit nerve injury-induced pain as quickly as analgesics, and the antalgic effect was similar to commonly used analgesics, such as morphine, and gabapentin. In this study, our group speculated that iMSCs-EVs may be useful in ameliorating chronic pelvic pain. As expected, EAP rats that received iMSCs-EVs presented with an improved nociceptive response to mechanical stimulation. Moreover, the upregulation of COX-2 was reversed, followed by a decrease in the production of prostaglandin E2. These data illuminated that iMSCs-EVs could ameliorate chronic pelvic pain by downregulating the overexpression of COX-2.

Although the pathogenesis of CP/CPPS is not completely untangled, growing evidence supports immune dysfunction and excessive expression of proinflammatory mediators in this process. It is well established that the imbalance of Th1/Th2 and Th17/Treg cells is strongly associated with the pathogenesis of CP/CPPS [13, 35]. The imbalance of Th1/Th2 is involved in the allergic disease and graft-versus-host disease and is related to autoimmune disorders [36]. It also has a role in the development of CP/CPPS. Owing to the increase in the number of Th1 cells, accompanied by the relative reduction in the number of Th2 cells, the proinflammatory mediator IFN-γ exhibited an excessive expression, and the opponent IL-4 showed an insufficient expression, which ultimately resulted in the destruction of glandular epithelial tissue and basal lamina [37]. Th17 and Treg cells are two classical types of CD4+ T cells subsets. Th17 cells and the secreted proinflammatory mediator IL-17A can cause autoimmunity and an inflammatory response. In contrast, Treg cells, and the secreted anti-inflammatory mediator IL-10 inhibit the autoimmune response and maintain immune homeostasis. Their opposite functions refer to a delicate balance between tolerance of autoimmunity and elicitation of autoimmunity [38]. The percentage of Th17 cells and the levels of IL-17A were found to increase in CP/CPPS patients [35] and rat EAP models.

Encouragingly, numerous studies have demonstrated that MSCs can inhibit the production of proinflammatory Th1 and Th17 cells, as well as facilitate the proliferation of immunosuppressive Treg cells, resulting in the suppression of ongoing inflammation. Furthermore, EVs released from MSCs have been reported to possess immunosuppressive and therapeutic capacities similar to those of MSCs themselves [39, 40]. For example, Nojehdehiet al [41]. reported that EVs released from adipose tissue-derived MSCs inhibited the proliferation of Th1 and Th17 cells and displayed anti-inflammatory effects in streptozotocin-induced type-1 autoimmune diabetes mice. Based on asthmatic mice, Du et al. [42] confirmed that EVs released from bone marrow-derived MSCs could obviously suppress chronic airway inflammation by facilitating the proliferation of IL-10-producing Treg cells. Similarly, in our study, we found that iMSCs-EVs administration decreased the percentages of Th1 and Th17 cells and downregulated the expression of IFN-γ and IL-17A, along with increases in Treg cells and IL-10. These data suggested that iMSCs-EVs administration was able to restore the imbalance of Th1/Th2 and Th17/Treg in the rat EAP model, which means that the balance of Th1/Th2 and Th17/Treg might be the therapeutic knots of iMSCs-EVs in the protective process against CP/CPPS. However, it remains unclear how iMSCs-EVs restore the imbalance of Th1/Th2 and Th17/Treg cells, and this problem will be explored in our next study.

## Conclusions

In sum, we demonstrated that iMSCs-EVs administration could ameliorate chronic pelvic pain, improve voiding dysfunction suppress inflammatory reactions, and promote prostatic tissue repair in CP/CPPS, which are mediated by downregulating the overexpression of COX-2 and restoring the imbalance of Th1/Th2 and Treg/Th17 cells. These data for the first time illuminate the therapeutic potential of iMSCs-EVs in treating CP/CPPS.

## Supplementary Information

**Additional file 1: Figure S1.** Expression of vimentin. A, DAPI staining identifies cell nuclei. B, Negative staining of vimentin in iMSCs. C, Merged image of A and B.

**Additional file 2: Figure S2.** Effect of iMSCs-EVs administration on the percentages of B cells in peripheral blood and spleen. A and B, Representative flow cytometric plots in each group. C and D, Statistical results indicated that no difference existed in the percentages of B cells among the three groups. Data are presented as the mean ± SD, *P<0.05, n.s. indicates no significance.

**Additional file 3: Figure S3.** Alterations of B cells in prostate tissues. A, Representative images of B cells. B, Statistical results indicated that no difference existed in the percentages of B cells among the three groups. Scale bar = 200 μm. Data are presented as the mean ± SD, *P<0.05, n.s. indicates no significance.

**Additional file 4: Figure S4.** Alterations of Th1 cells and Th2 cells in prostate tissues. A and C, Representative images of Th1 cells and Th2 cells. B, Statistical results indicated that iMSCs-EVs administration could reverse the increases in the percentages of Th1 cells. D, Statistical results indicated that no difference existed in the numbers of Th2 cells among the three groups. Scale bar = 200 μm. Data are presented as the mean ± SD, *P<0.05, n.s. indicates no significance.

**Additional file 5: Figure S5.** Alterations of Th17 cells and Treg cells in prostate tissues. A and C, Representative images of Th17 cells and Treg cells. B, Statistical results indicated that iMSCs-EVs administration could increase the percentages of Treg cells. D, Statistical results indicated that iMSCs-EVs administration could reverse the increases in the percentages of Th17 cells. Scale bar = 200 μm. Data are presented as the mean ± SD, *P<0.05, n.s. indicates no significance.

## Data Availability

The datasets used and/or analyzed during the current study are available from the corresponding author upon reasonable request.

## Abbreviations

hiPSCs: Human induced pluripotent stem cells
MSCs: Mesenchymal stem cells
CP/CPPS: Chronic prostatitis/chronic pelvic pain syndrome
EVs: Extracellular vesicles
iMSCs-EVs: Extracellular vesicles released from mesenchymal stem cells derived from human induced pluripotent stem cells
EAP: Experimental autoimmune prostatitis
COX-2: Cyclooxygenase-2
CM: Conditioned medium
PBS: Phosphate-buffered saline
TEM: Transmission electron microscopy
PAHS: Prostate antigen homogenate supernatant
IFN-γ: Interferon-γ
IL-2: Interleukin-2
IL-4: Interleukin-4
IL-6: Interleukin-6
IL-10: Interleukin-10
IL-17A: interleukin 17A
ELISA: Enzyme-linked immunosorbent assay
H&E: Hematoxylin and eosin
PVDF: Polyvinylidene difluoride
SD: Standard deviation
ANOVA: One-way analysis of variance
