# Supplementary figures and images for: Extracellular vesicles released from hiPSC-derived MSCs attenuate chronic prostatitis/chronic pelvic pain syndrome in rats by immunoregulation
Source: Stem Cell Res Ther. 2021 Mar 20;12:198. doi: 10.1186/s13287-021-02269-x (PMC7981875; doi:10.1186/s13287-021-02269-x)

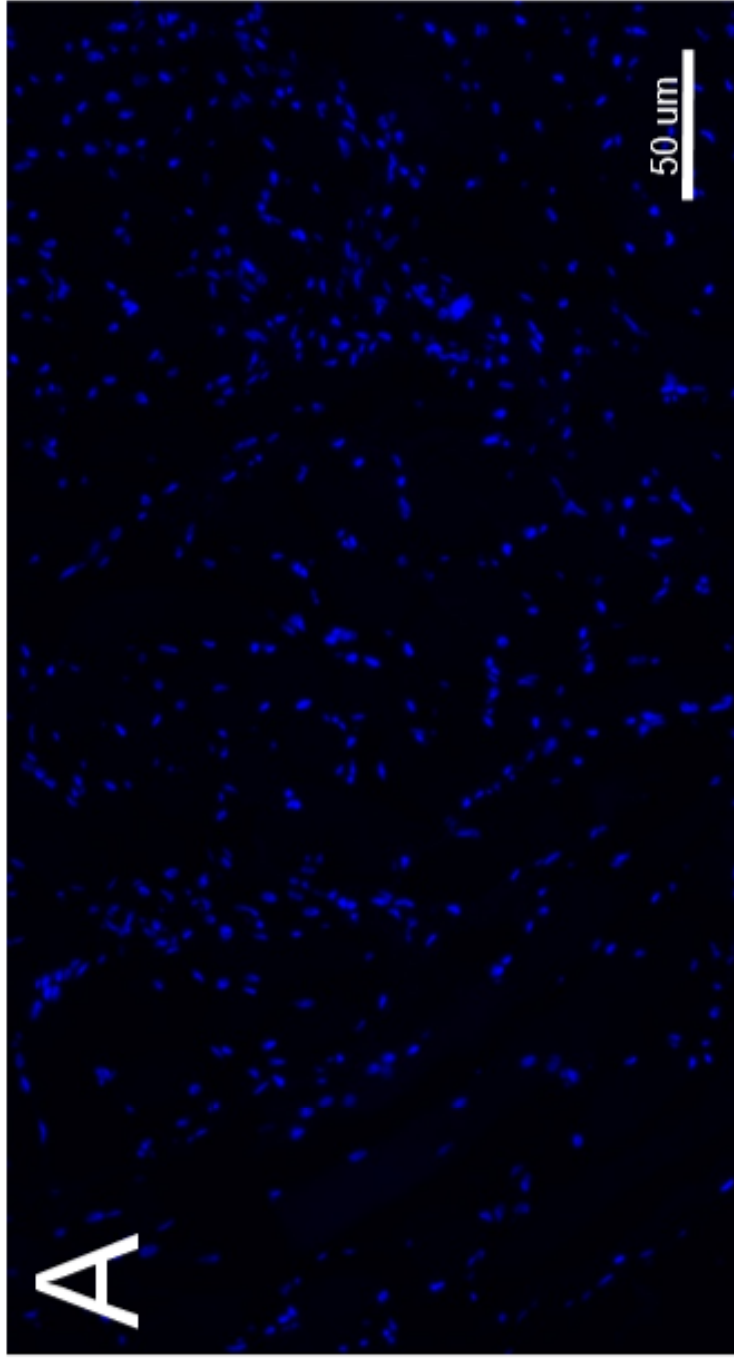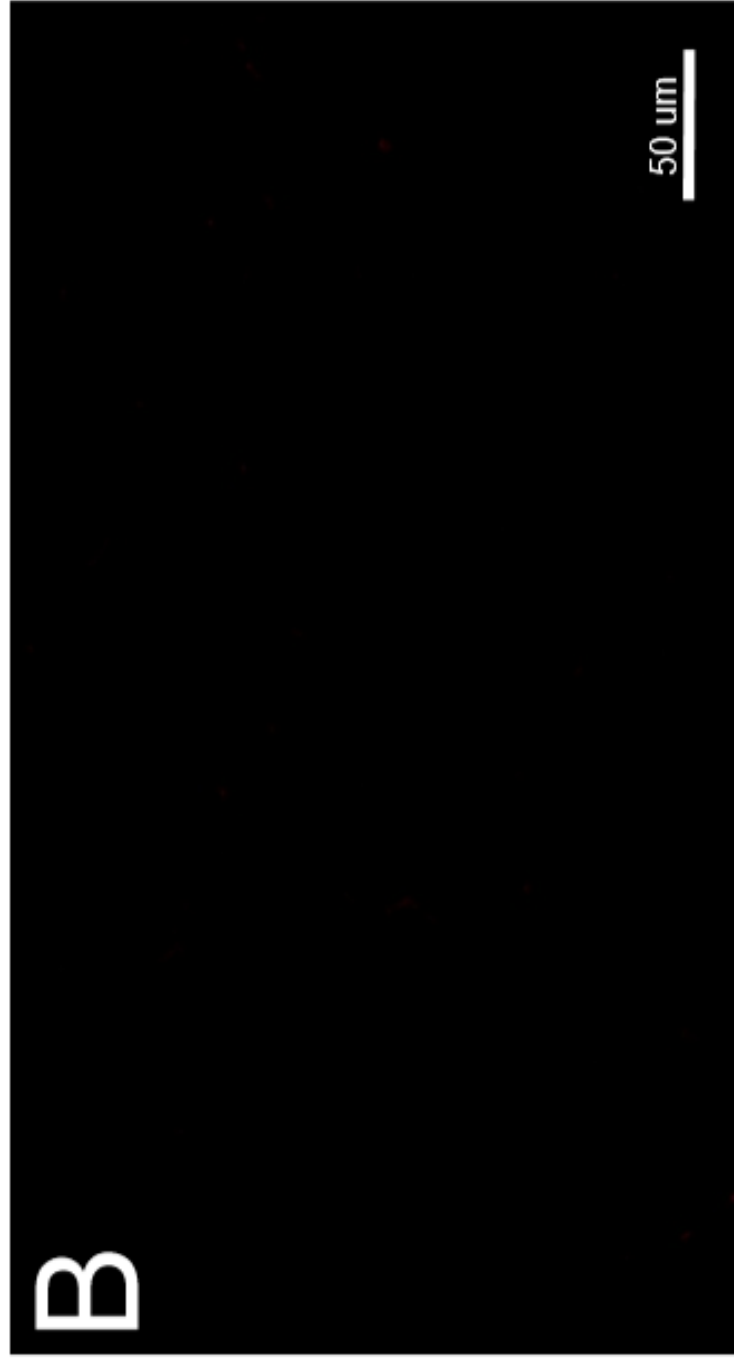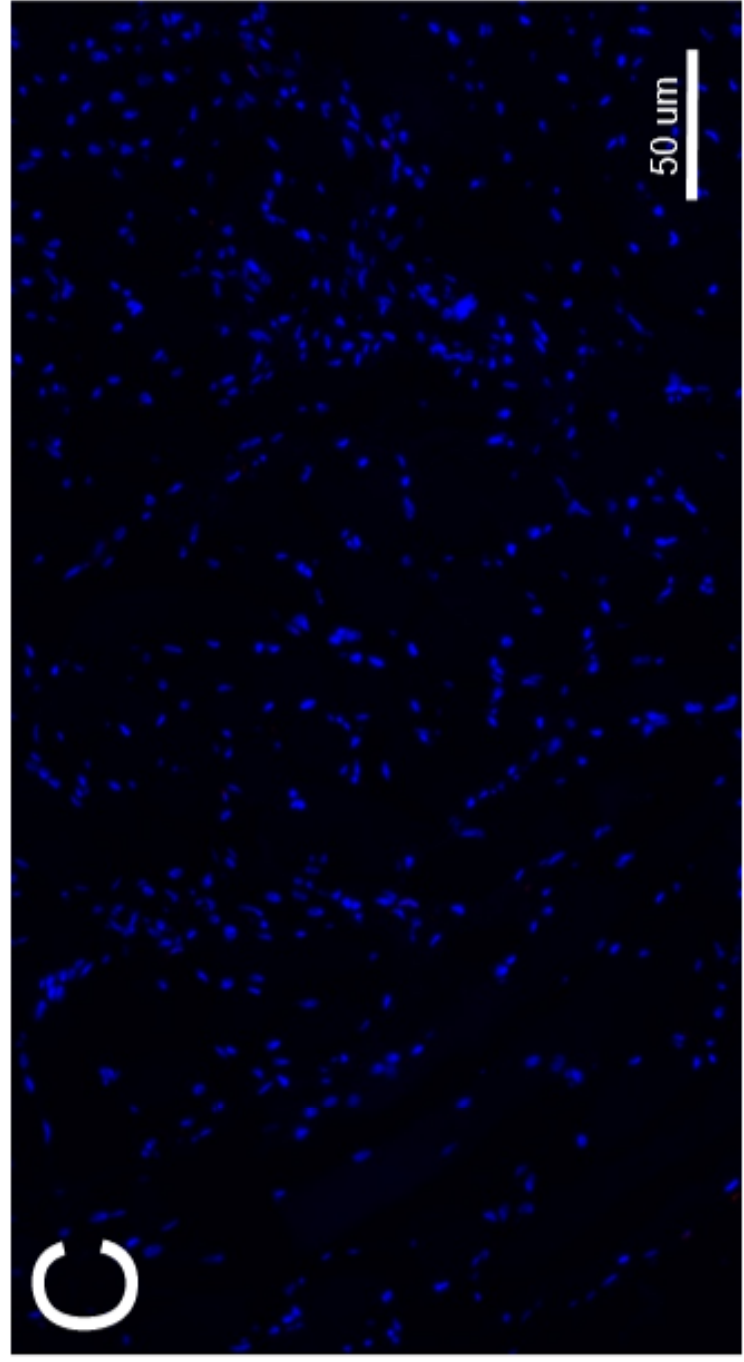

Supplement: Supplementary file 1 — Additional file 1: Figure S1. Expression of vimentin. A, DAPI staining identifies cell nuclei. B, Negative staining of vimentin in iMSCs. C, Merged image of A and B. [file 13287_2021_2269_MOESM1_ESM.pdf]

A

Blood

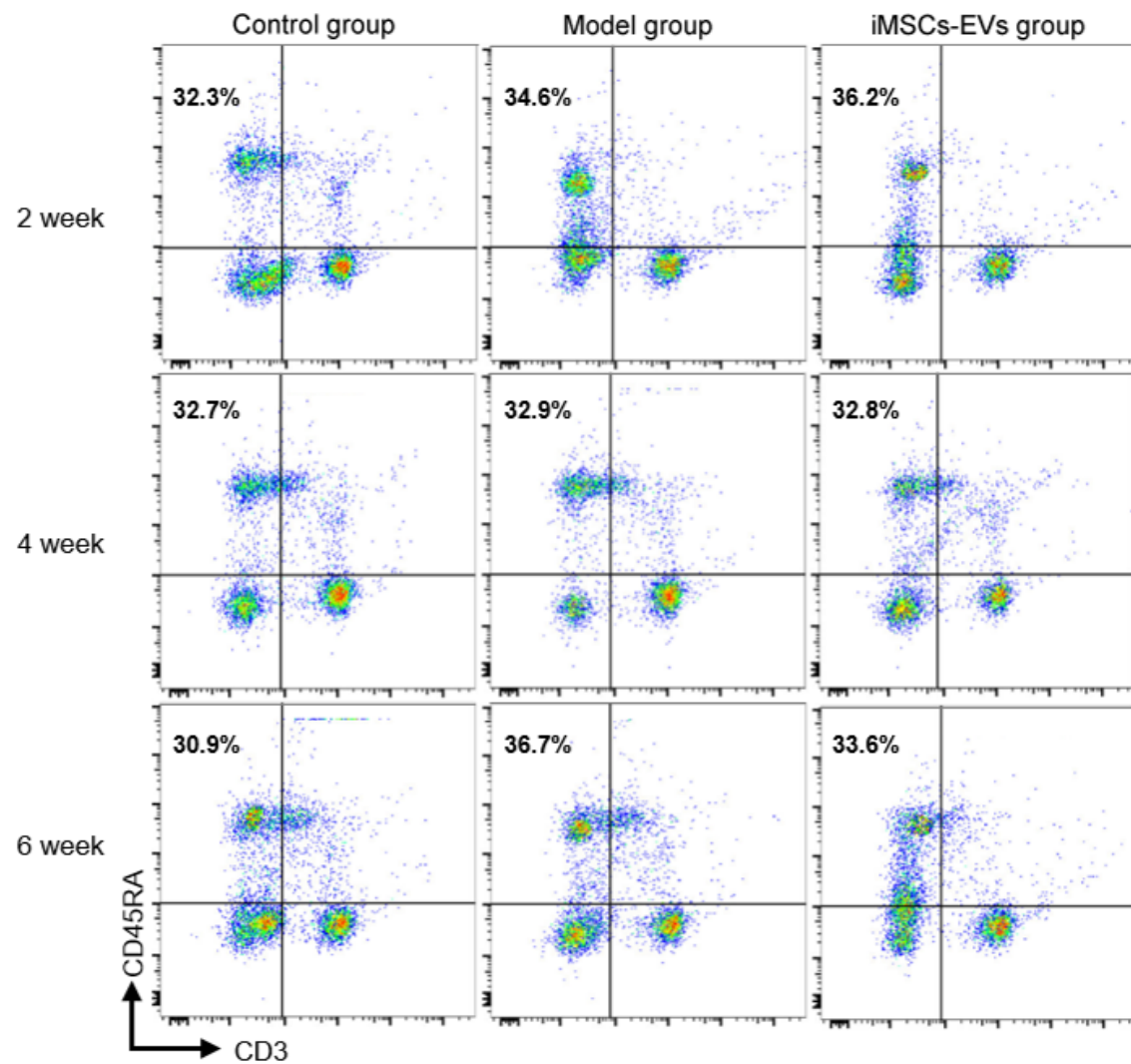

B

Spleen

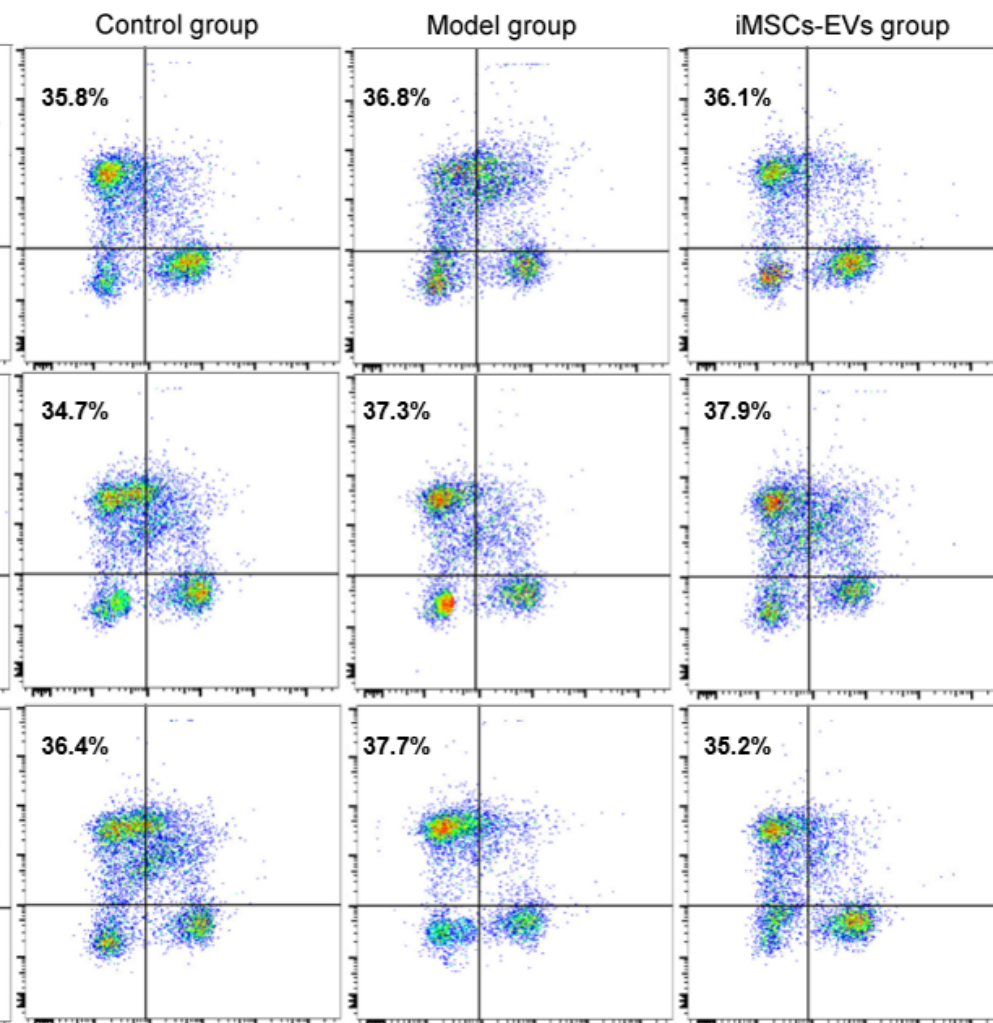

C

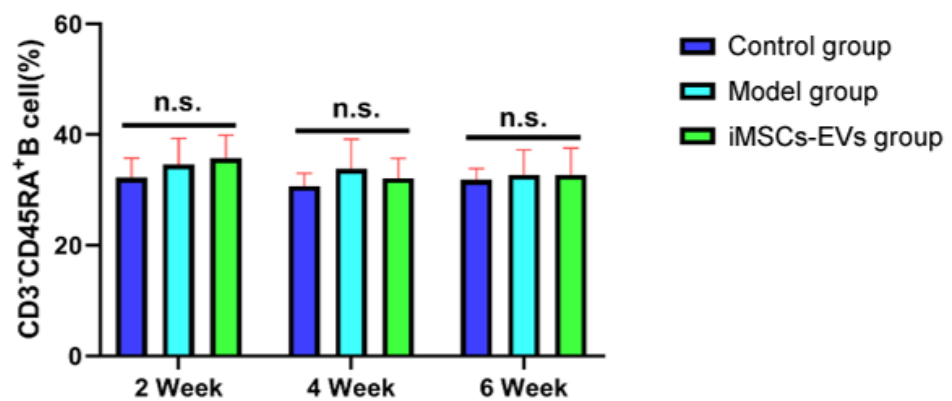

D

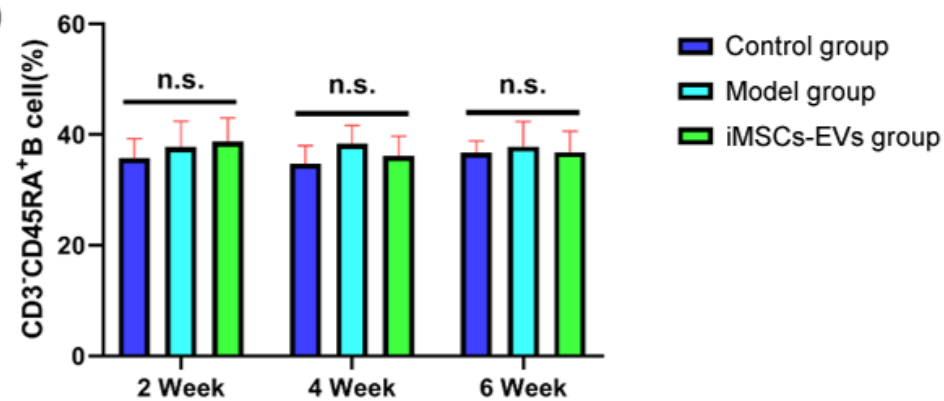

Supplement: Supplementary file 2 — Additional file 2: Figure S2. Effect of iMSCs-EVs administration on the percentages of B cells in peripheral blood and spleen. A and B, Representative flow cytometric plots in each group. C and D, Statistical results indicated that no difference existed in the percentages of B cells among the three groups. Data are presented as the mean ± SD, *P<0.05, n.s. indicates no significance. [file 13287_2021_2269_MOESM2_ESM.pdf]

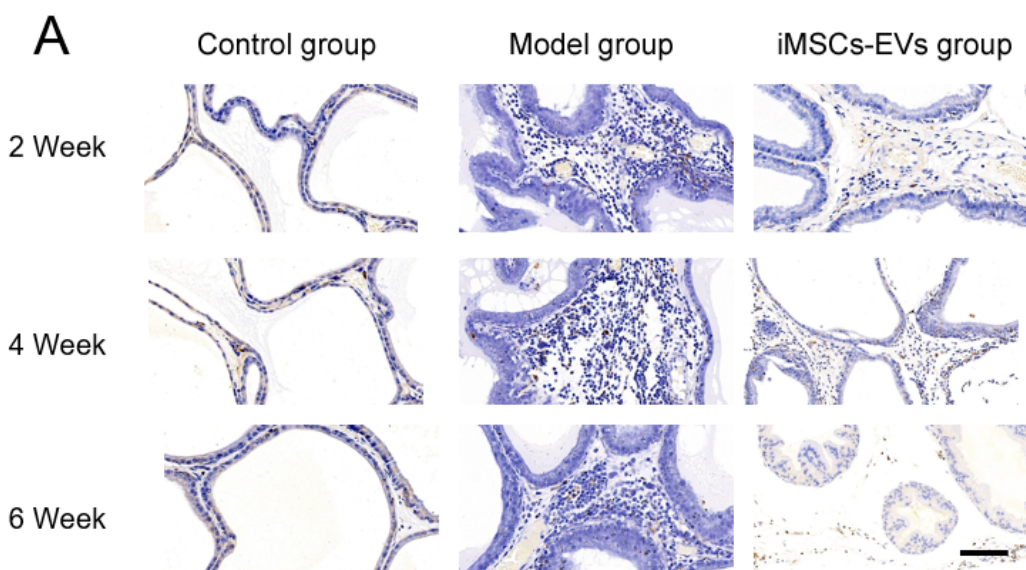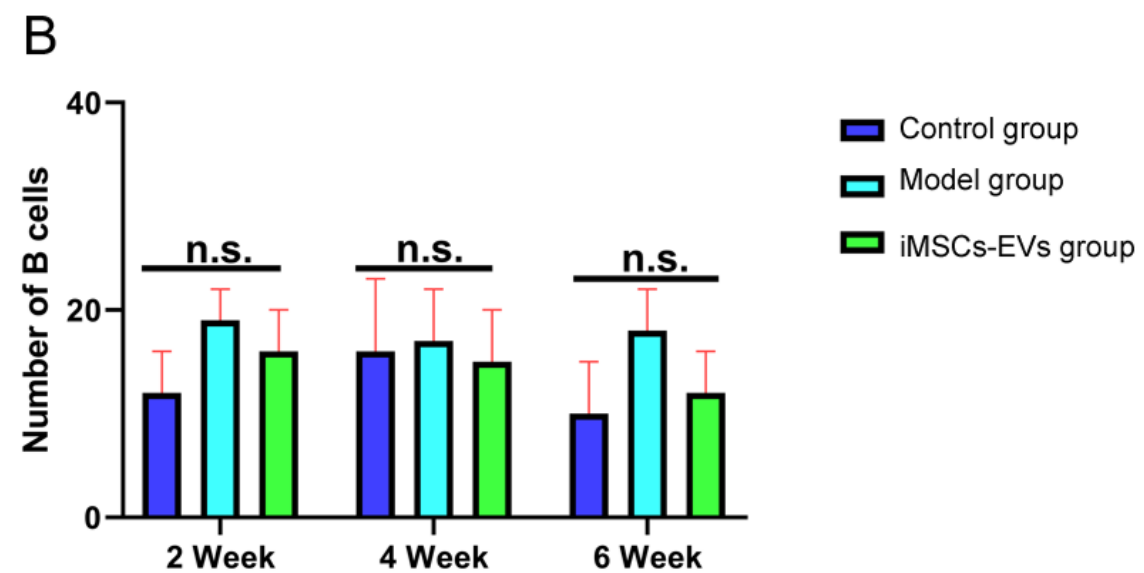

Supplement: Supplementary file 3 — Additional file 3: Figure S3. Alterations of B cells in prostate tissues. A, Representative images of B cells. B, Statistical results indicated that no difference existed in the percentages of B cells among the three groups. Scale bar = 200 μm. Data are presented as the mean ± SD, *P<0.05, n.s. indicates no significance. [file 13287_2021_2269_MOESM3_ESM.pdf]

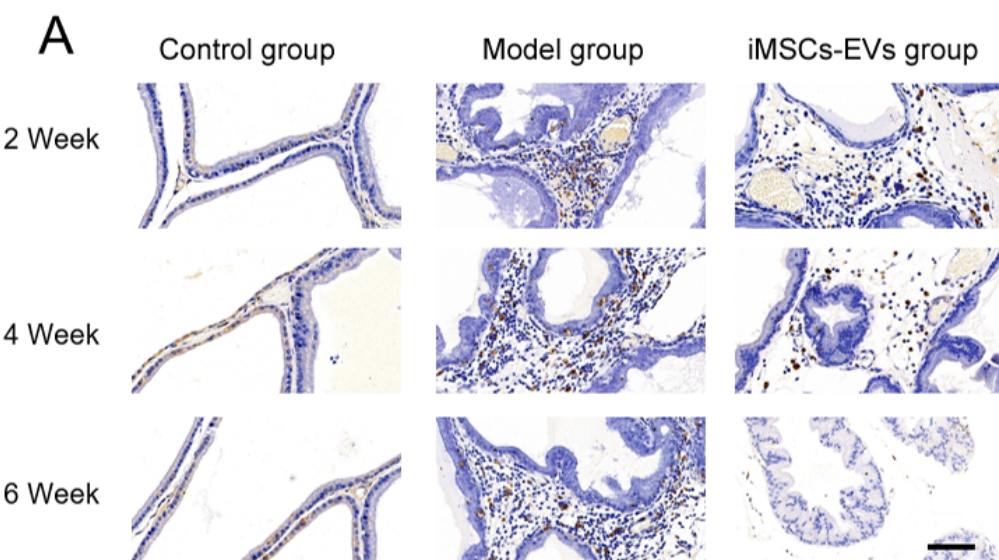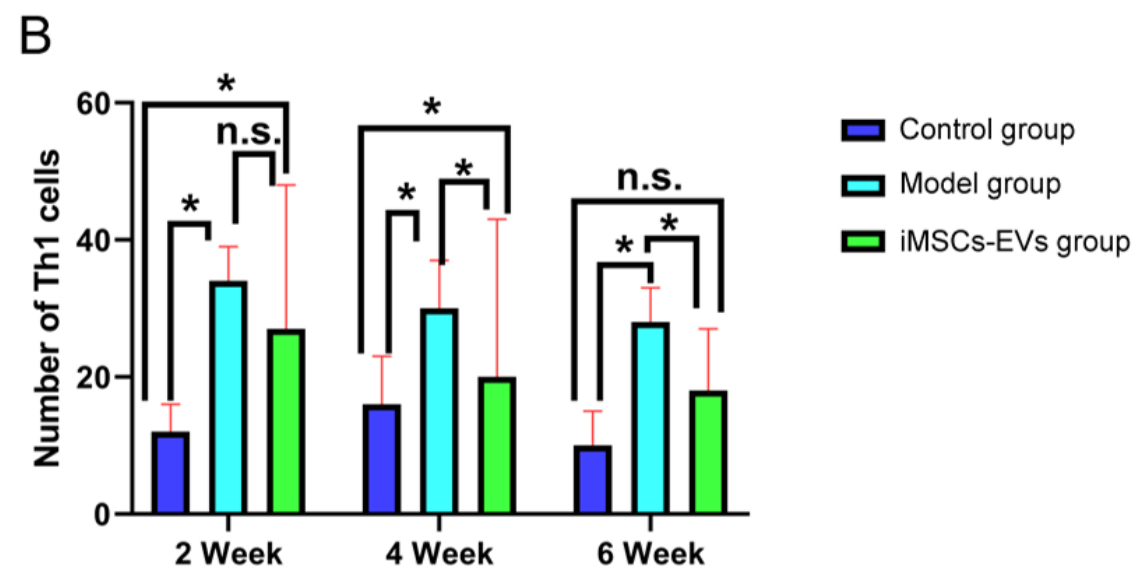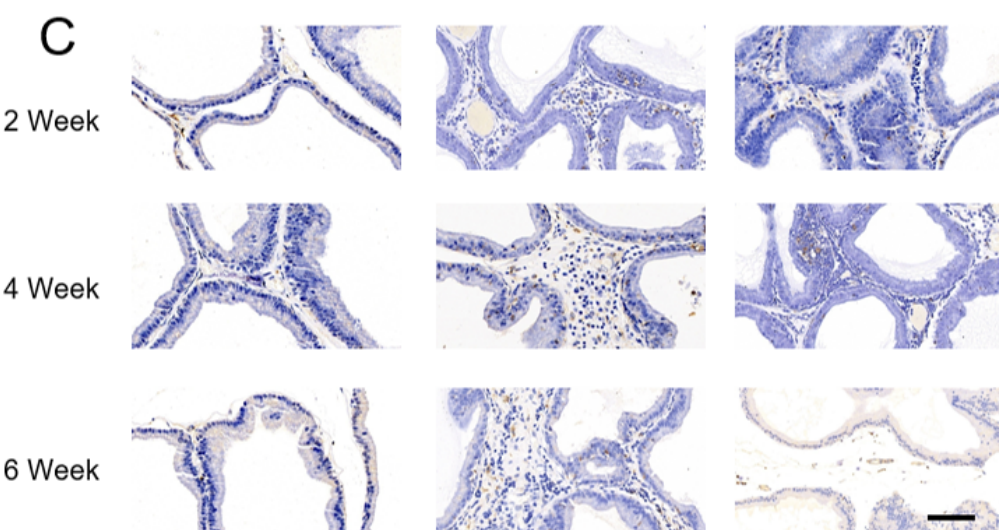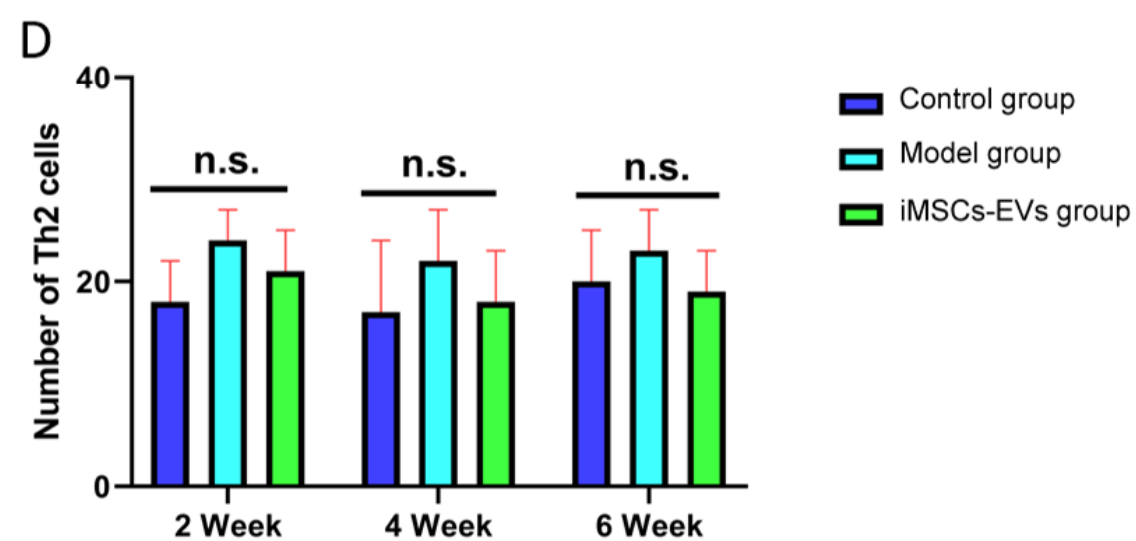

Supplement: Supplementary file 4 — Additional file 4: Figure S4. Alterations of Th1 cells and Th2 cells in prostate tissues. A and C, Representative images of Th1 cells and Th2 cells. B, Statistical results indicated that iMSCs-EVs administration could reverse the increases in the percentages of Th1 cells. D, Statistical results indicated that no difference existed in the numbers of Th2 cells among the three groups. Scale bar = 200 μm. Data are presented as the mean ± SD, *P<0.05, n.s. indicates no significance. [file 13287_2021_2269_MOESM4_ESM.pdf]

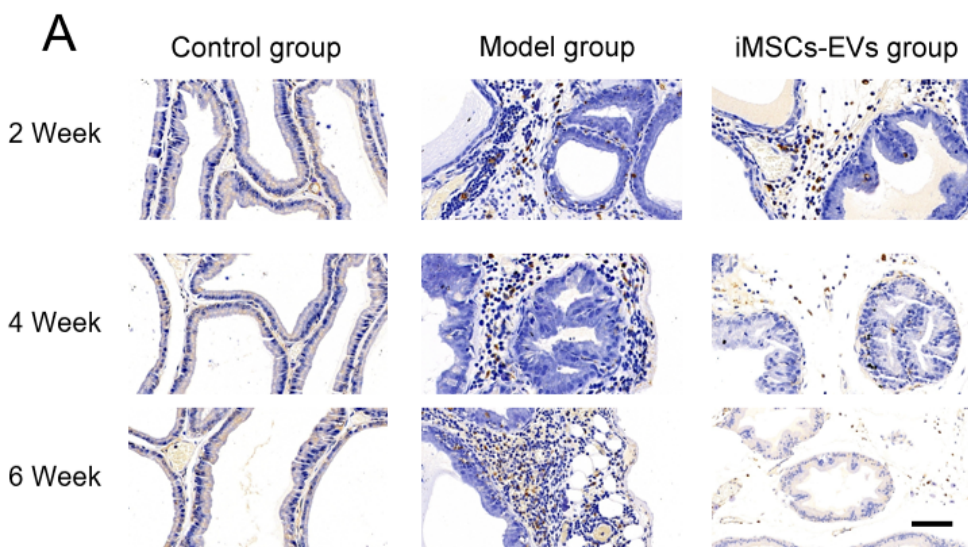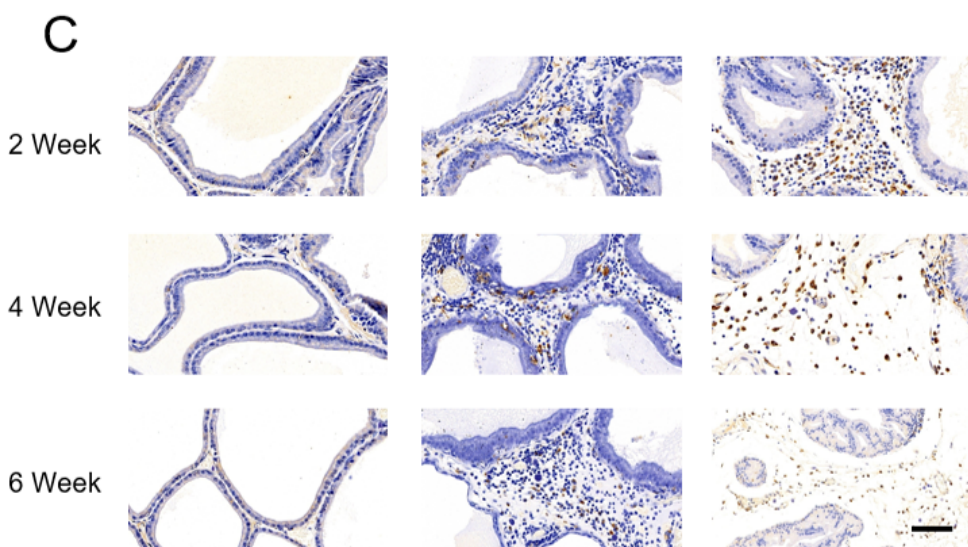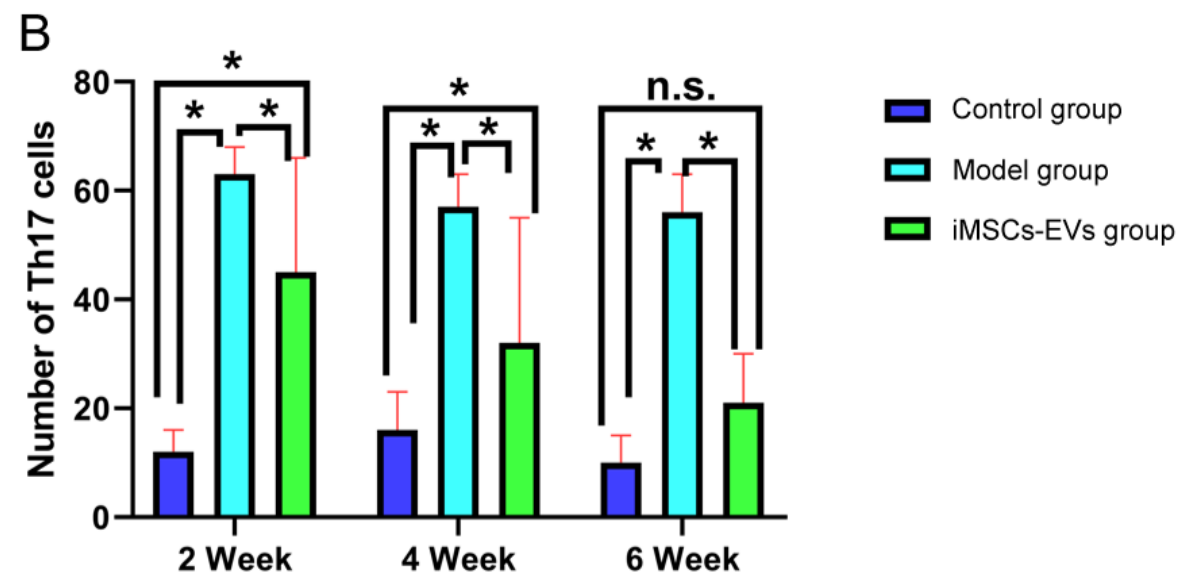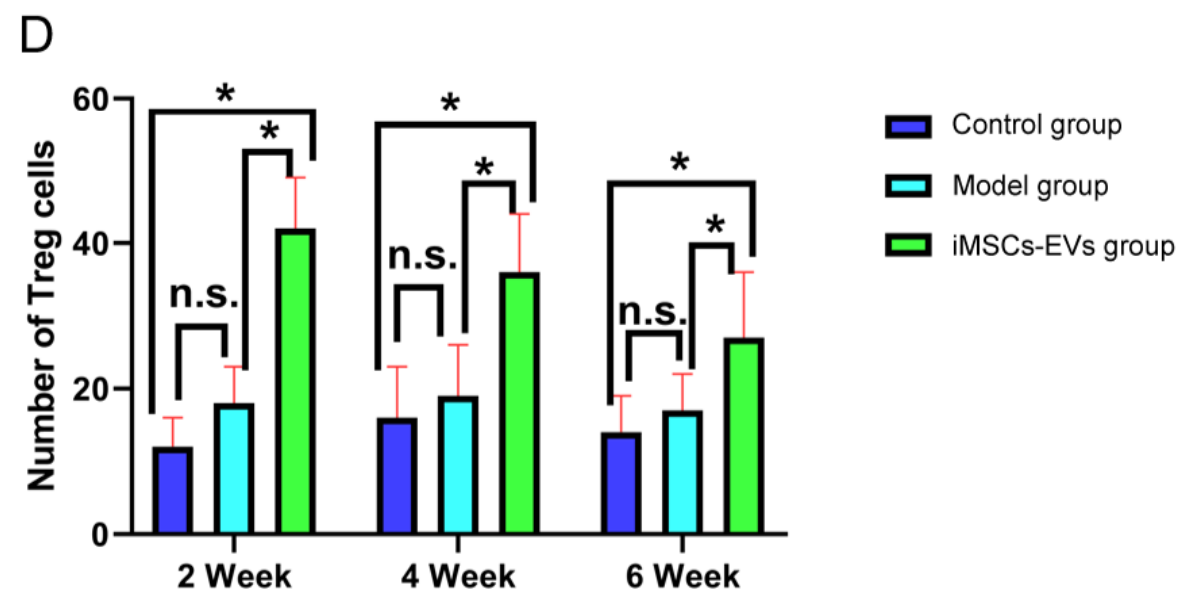

Supplement: Supplementary file 5 — Additional file 5: Figure S5. Alterations of Th17 cells and Treg cells in prostate tissues. A and C, Representative images of Th17 cells and Treg cells. B, Statistical results indicated that iMSCs-EVs administration could increase the percentages of Treg cells. D, Statistical results indicated that iMSCs-EVs administration could reverse the increases in the percentages of Th17 cells. Scale bar = 200 μm. Data are presented as the mean ± SD, *P<0.05, n.s. indicates no significance. [file 13287_2021_2269_MOESM5_ESM.pdf]
